# Alteration of cortical functional networks in mood disorders with resting-state electroencephalography

**DOI:** 10.1038/s41598-022-10038-w

**Published:** 2022-04-08

**Authors:** Sungkean Kim, Ji Hyun Baek, Se-hoon Shim, Young Joon Kwon, Hwa Young Lee, Jae Hyun Yoo, Ji Sun Kim

**Affiliations:** 1 Department of Human-Computer Interaction, Hanyang University, Ansan, Republic of Korea; 2 Department of Psychiatry, Samsung Medical Center, Seoul, Republic of Korea; 3 Department of Psychiatry, Soonchunhyang University Cheonan Hospital, Cheonan, Republic of Korea; 4 Department of Psychiatry, Seoul St. Mary’s Hospital, College of Medicine, The Catholic University of Korea, Seoul, Republic of Korea

**Keywords:** Computational neuroscience, Diagnostic markers, Psychiatric disorders

## Abstract

Studies comparing bipolar disorder (BD) and major depressive disorder (MDD) are scarce, and the neuropathology of these disorders is poorly understood. This study investigated source-level cortical functional networks using resting-state electroencephalography (EEG) in patients with BD and MDD. EEG was recorded in 35 patients with BD, 39 patients with MDD, and 42 healthy controls (HCs). Graph theory-based source-level weighted functional networks were assessed via strength, clustering coefficient (CC), and path length (PL) in six frequency bands. At the global level, patients with BD and MDD showed higher strength and CC, and lower PL in the high beta band, compared to HCs. At the nodal level, compared to HCs, patients with BD showed higher high beta band nodal CCs in the right precuneus, left isthmus cingulate, bilateral paracentral, and left superior frontal; however, patients with MDD showed higher nodal CC only in the right precuneus compared to HCs. Although both MDD and BD patients had similar global level network changes, they had different nodal level network changes compared to HCs. Our findings might suggest more altered cortical functional network in patients with BD than in those with MDD.

## Introduction

Bipolar disorder (BD) and major depressive disorder (MDD) are considered representative mood disorders. MDD is a debilitating disease that includes depressed mood, diminished interests, impaired cognition, vegetative symptoms, and changed psychomotor activity^1^. When MDD occurs with an individual who has also had a history of manic episode, this is called BD^2^. BD is a chronic and complex disorder of mood that is characterized by a combination of manic, hypomanic, and depressive episodes with subsyndromal symptoms extant between the mood episodes^3^. These two mood disorders were the leading causes of worldwide disability and morbidity^3^. These two psychiatric conditions exhibit similar severe depressive symptoms and show no difference in the duration of affective episodes during the course of illness^4^. These two mood disorders are very different, but they have similarities. Therefore, the similarities and differences of the two mood conditions have been of interest to clinicians.

Previous neuroimaging studies demonstrated widespread brain structural and functional alterations in both BD and MDD, such as the prefrontal cortex, limbic system, ventral striatum, and thalamus^5–8^. A study investigating the volume of gray matter in affective disorders found decreased gray matter volume in the medial frontal cortex and anterior cingulate cortex, specifically in MDD^9^. Strakowsk et al.^10^ observed a significant difference between patients with BD and healthy volunteers in the volumes of amygdala, thalamus, pallidum, and striatum belonging to the structural network that putatively modulate human mood. For functional neuroimaging studies, one previous study showed that BD and MDD patients displayed significant decreased activity in the paracentral lobule, precuneus, and paracingulate gyrus within bilateral hemisphere, and the postcentral gyrus and precentral gyrus within right hemisphere^11^. However, studies on direct comparisons between BD and MDD, i.e. including and handling both diseases in one study, are sparse, with inconclusive results.

To date, most of the previous studies have been performed using fMRI, which is a suitable imaging tool to investigate regional brain information owing to its excellent spatial resolution. However, fMRI lacks temporal resolution to elucidate neural processes occurring over the course of milliseconds^12^. Electroencephalography (EEG) is an appropriate tool to address the limitations of fMRI with high temporal resolution^13^. In addition, EEG is sensitive to alterations in neurotransmission secondary to pharmacological manipulations or brain dysfunction^14^. Resting-state brain activity reflects the baseline status of the brain and has been proposed for a means of exploring the underlying pathophysiology of mental disorders^15^. Thus, resting-state EEG analysis, which has been validated for utilization as a tool^16^, could help to better understand the pathophysiology of mental disorders.

Recently, an increasing number of researchers have studied cortical functional networks based on graph theory^17,18^. Graph theory-based brain network analysis could assist to comprehend the brain mechanisms of psychiatric disorders, such as mood disorders. However, to date, only a few studies have examined the resting-state cortical functional network in patients with BD^19,20^ or MDD^21–23^ using graph theory. Furthermore, limited studies have included both BD and MDD and compared their networks. One of the studies comparing BD and MDD demonstrated that both disorders showed similar network changes with decreased CC and efficiency, and increased PL within the default-mode network (DMN) and limbic system network^24^. Particularly, no study has investigated and compared their networks using EEG. Although BD and MDD are distinct mood disorders, they exhibit similar severe depressive symptoms and have similar depressive courses^4^. Despite the clinical characteristics, the similarities and differences in brain functional networks between BD and MDD have been poorly understood. Therefore, further studies are needed to explore and compare their networks.

EEG has been known to lack spatial resolution coming from volume conduction^25,26^ and poor signal-to-noise ratio^27,28^. However, source imaging methods, advantageous alternatives to prevent the issue, can considerably enhance the spatial resolution of EEG. The objective of the current study was to investigate and compare cortical functional networks from resting-state EEG in patients with BD and MDD via source-level weighted network analysis. We also explored the associations between cortical network properties and psychological measures in patients with BD and MDD. We hypothesized that patients with BD and MDD would display altered cortical functional networks compared to healthy controls (HCs) and that two patient groups would show different degrees of alteration in their networks.

## Results

### Demographic and psychological characteristics

Table 1 presents the comparisons of demographic and psychological characteristics among the patients with BD and MDD and HCs. There was a significant difference in education years (*p* < 0.001). HCs showed significantly higher education years than patients with BD and MDD. In addition, there were significant differences in STAI state, STAI trait, and BDI (STAI state: *p* < 0.001; STAI trait: *p* < 0.001; BDI: *p* < 0.001). HC presented significantly lower STAI state, STAI trait, and BDI scores than patients with BD and MDD.

**Table 1:** Demographic characteristics of all study participants.

	BD (N = 35)	MDD (N = 39)	HC (N = 42)	*P*	Post-hoc (Bonferroni)
Age (years)	32.57 ± 11.61	31.62 ± 12.28	29.43 ± 5.95	0.380	
**Sex**				0.412	
Male	14 (40.0)	20 (51.3)	23 (54.8)		
Female	21 (60.0)	19 (48.7)	19 (45.2)		
Education (years)	12.97 ± 2.53	11.87 ± 1.94	15.33 ± 2.19	< 0.001	BD < HC, MDD < HC
Onset age (years)	25.34 ± 9.79	29.44 ± 11.83		0.112	
Duration of illness (years)	7.26 ± 7.71	2.56 ± 2.73		0.001	
STAI state	60.69 ± 9.54	64.85 ± 7.60	31.07 ± 6.85	< 0.001	BD > HC, MDD > HC
STAI trait	62.06 ± 9.91	64.95 ± 5.61	33.76 ± 7.20	< 0.001	BD > HC, MDD > HC
BDI	47.66 ± 9.88	47.74 ± 10.80	23.38 ± 3.93	< 0.001	BD > HC, MDD > HC

BD, bipolar disorder; MDD, major depressive disorder; HC, healthy control; STAI, State-Trait Anxiety Inventory; BDI, Beck Depression Inventory.

### Global level differences in cortical functional networks

Table 2 presents the comparisons of global level indices among the BD, MDD, and HC groups. There were significant differences in the three global level indices of the high beta band. The strength (*p* = 0.001, η^2^ = 0.112) and CC (*p* = 0.001, η^2^ = 0.114) of the high beta band were significantly higher in patients with BD and MDD than in HCs. In contrast, the PL of the high beta band was significantly lower in patients with BD and MDD than in HCs (*p* < 0.001, η^2^ = 0.129). There was no significant difference between the patient groups for the three network indices of the high beta band. Furthermore, there was no significant difference among the three groups in the other frequency bands. Mean weighted matrices from phase-locking values (PLVs) for the high beta band in each group and pair-wise comparisons were presented in the supplementary results.

**Table 2:** Mean and standard deviation values of global network indices including strength, clustering coefficient (CC), and path length (PL) in each frequency band among the bipolar disorder, major depressive disorder, and healthy control groups.

	BD (N = 35)	MDD (N = 39)	HC (N = 42)	Effect size (η^2^)	P*	Post-hoc (Bonferroni)
**Delta band**						
Strength	28.02 ± 1.26	27.99 ± 1.12	27.67 ± 0.89	0.006	0.702	
CC	0.41 ± 0.02	0.41 ± 0.02	0.41 ± 0.01	0.006	0.728	
PL	2.57 ± 0.10	2.57 ± 0.09	2.59 ± 0.07	0.004	0.802	
**Theta band**						
Strength	24.51 ± 1.40	24.28 ± 1.09	23.91 ± 0.87	0.023	0.274	
CC	0.36 ± 0.02	0.35 ± 0.02	0.35 ± 0.01	0.022	0.282	
PL	3.02 ± 0.15	3.04 ± 0.12	3.07 ± 0.10	0.019	0.344	
**Alpha band**						
Strength	26.02 ± 2.27	26.97 ± 2.99	25.06 ± 2.00	0.037	0.124	
CC	0.38 ± 0.03	0.39 ± 0.04	0.36 ± 0.03	0.036	0.127	
PL	2.84 ± 0.24	2.75 ± 0.29	2.94 ± 0.21	0.031	0.173	
**Low beta band**						
Strength	21.22 ± 1.08	21.03 ± 0.91	20.40 ± 0.93	0.077	0.012	
CC	0.30 ± 0.02	0.30 ± 0.01	0.29 ± 0.01	0.077	0.011	
PL	3.55 ± 0.17	3.59 ± 0.14	3.68 ± 0.15	0.080	0.009	
**High beta band**						
Strength	17.54 ± 1.14	17.31 ± 1.02	16.54 ± 0.85	0.112	**0.001**	BD > HC, MDD > HC
CC	0.25 ± 0.02	0.24 ± 0.01	0.23 ± 0.01	0.114	**0.001**	BD > HC, MDD > HC
PL	4.38 ± 0.30	4.44 ± 0.25	4.67 ± 0.24	0.129	** < 0.001**	BD < HC, MDD < HC
**Gamma band**						
Strength	14.18 ± 1.34	14.29 ± 1.28	13.96 ± 1.02	0.017	0.383	
CC	0.19 ± 0.02	0.19 ± 0.02	0.19 ± 0.01	0.017	0.386	
PL	5.29 ± 0.55	5.24 ± 0.46	5.38 ± 0.43	0.015	0.429	

*The *p *value was adjusted via Bonferroni correction with 0.05/18 = 0.002778.

BD, bipolar disorder; MDD, major depressive disorder; HC, healthy control.

### Nodal level differences in cortical functional networks

**Figure. 1:**
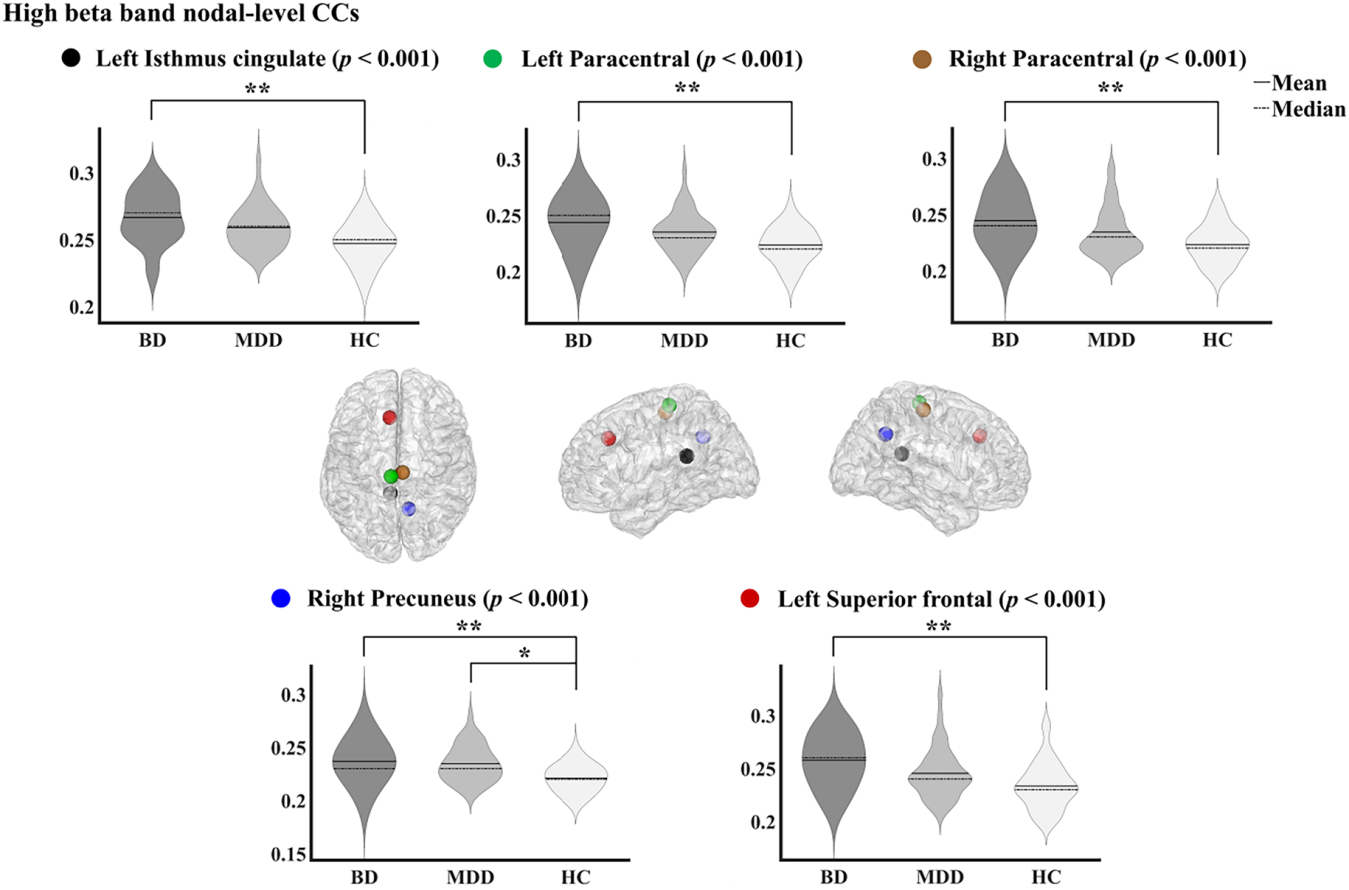
Violin plots of significant nodal clustering coefficients in high beta band for bipolar disorder, major depressive disorder, and healthy controls. The *p* value was adjusted via Bonferroni correction. CC, clustering coefficient; BD, bipolar disorder; MDD, major depressive disorder; HC, healthy control. *p < 0.05 (corrected), **p < 0.01 (corrected).

On the basis of the significant difference in the global high beta band CC among the three groups, we decided to explore possible differences at the nodal level in the high beta band. Figure 1 shows the violin plots of the significant nodal CCs in five regions among the three groups. The nodal CCs of the BD and MDD groups were significantly higher than that of HCs in one region (right precuneus, *p* < 0.001, η^2^ = 0.126). The nodal CCs of the BD were significantly higher than those of HCs in four regions (left isthmus cingulate: *p* < 0.001, η^2^ = 0.144; left paracentral: *p* < 0.001, η^2^ = 0.131; right paracentral: *p* < 0.001, η^2^ = 0.125; left superior frontal: *p* < 0.001, η^2^ = 0.132).

### Correlation between network indices and psychological characteristics

**Figure. 2:**
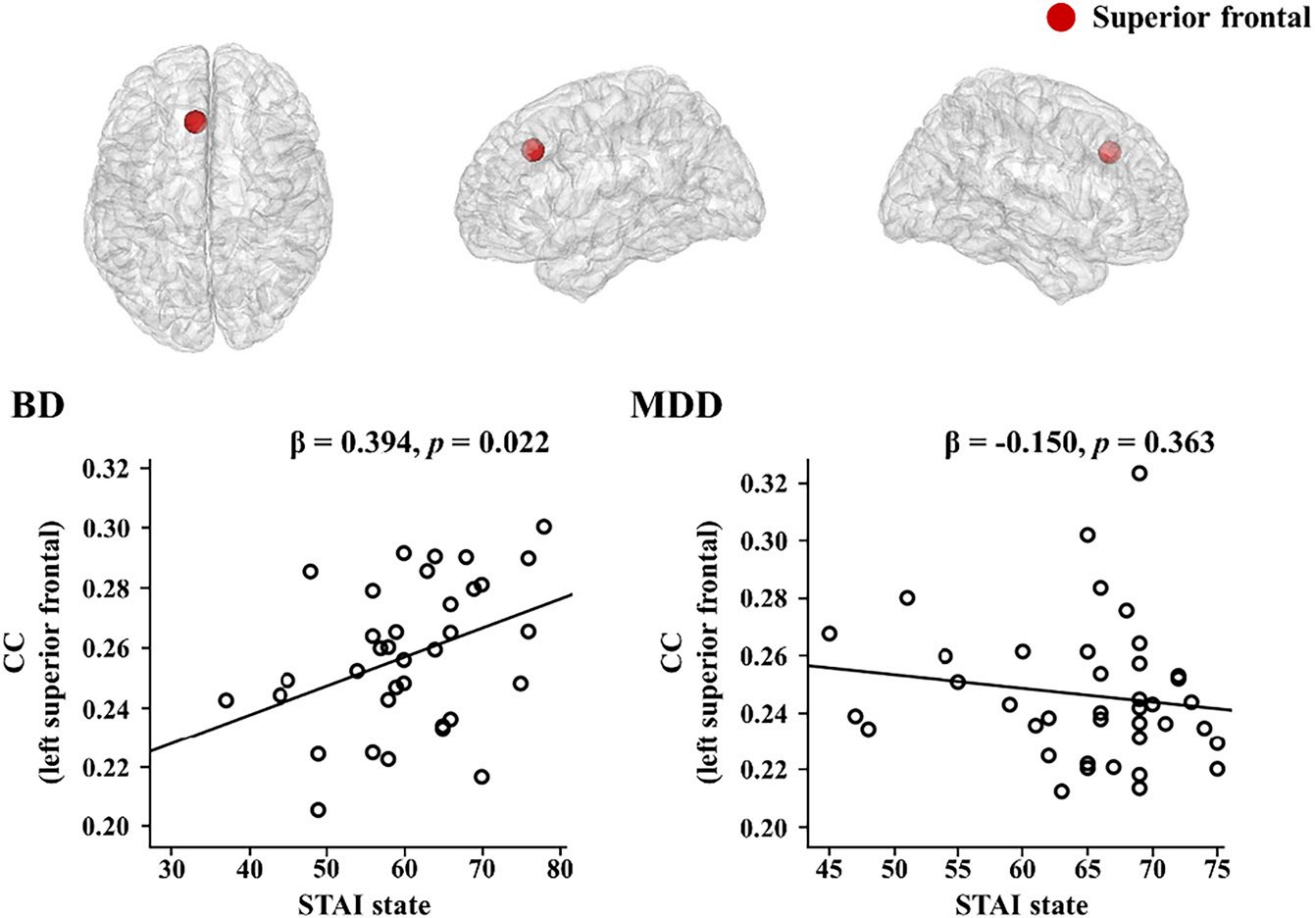
Correlations between nodal clustering coefficients (CC) and psychological measures in high beta band for patient groups. BD, bipolar disorder; MDD, major depressive disorder; STAI, State-Trait Anxiety Inventory.

The correlations between the network indices and psychological measures were evaluated in the high beta band. Following the 5000 bootstrap resampling technique, the two patient groups only showed a significantly different association between the nodal CC in the left superior frontal region and the STAI state. A post-hoc analysis of the significant interaction term (R^2^ = 0.134, F = 2.660, df = 69, *p* = 0.040) showed an association between the nodal CC in the left superior frontal region and the STAI state in patients with BD (β = 0.394, p = 0.022) but not in patients with MDD (β = -0.150, p = 0.363), a pattern observed after considering duration of illness (Fig. 2).

## Discussion

This study investigated cortical functional networks during resting-state EEG in patients with BD and MDD and HCs. We observed significant differences among these groups in the high beta band. First, at the global level, strength and CC were significantly higher, while PL was lower in both patient groups compared with HCs. Second, at the nodal level, the CC in the right precuneus was significantly higher in both patient groups than that in HCs. Additionally, patients with BD showed higher CCs in the left isthmus cingulate, bilateral paracentral, and left superior frontal, compared with HCs. Third, the nodal level high beta band CCs of the left superior frontal region correlated with the STAI state in patients with BD.

Although only few studies have explored graph theory-based network properties in BD and MDD, they have been documented to have abnormal brain connectivity at the network-level. Structural brain networks from diffusion tensor imaging exhibited lower CC and efficiency, and longer PL in BD^29,30^. Kim et al.^19^ showed decreased CC and efficiency, and increased PL in the alpha band during resting-state EEG in patients with BD. Another resting-state EEG study demonstrated that BD showed higher strength and CC, and lower PL in the theta band^20^. Furthermore, several neuroimaging studies have shown altered functional brain networks in MDD patients. fMRI studies observed greater efficiency and lower PL and CC in MDD patients^21,31,32^. Guo et al.^33^ reported that patients with MDD had stronger efficiency and shorter PL than HCs, but no difference was found at the local level between the MDD and HC groups. In addition, a resting-state EEG study^23^ indicated increased beta band global efficiency in patients with MDD, while nodal level efficiency in patients with MDD was almost the same as that in HCs. One resting-state fMRI study^24^ comparing BD and MDD patients demonstrated similar network changes in both patients with decreased CC and efficiency, and increased PL compared to HCs within the DMN and limbic system network. Although the previous results tend to be inconsistent regarding the direction of altered network properties in the disorders, these findings support our results that patients with BD and MDD displayed similar abnormal cortical functional networks at the global level.

Although previous studies did not show a consensus of abnormality in a specific frequency band in patients with BD and MDD, various studies have reported that the beta band is closely related to the patients. Several EEG studies have concluded that beta power positively correlates with the DMN activity in BD^34–36^. A recent magnetoencephalographic (MEG) study^37^ revealed that the limbic network connectivity in the beta band could be a good objective biomarker for BD. In terms of MDD, one EEG study^38^ revealed that patients with MDD showed greater beta band functional connectivity within the DMN. In addition, MEG studies have reported the association of altered connectivity in the beta band with MDD^39,40^. Excessive beta band synchronization, which is associated with maintaining the brain’s status quo, might be a mechanism driving brain inflexibility in BD and MDD patients. Previous studies back up our findings that excessive neural processing in the beta band might be associated with the pathophysiology in depressive states in mood disorders.

At the nodal level, our results showed that compared to HCs, patients with BD showed higher nodal CCs of the high beta band in the right precuneus, left isthmus cingulate, bilateral paracentral, and left superior frontal. In contrast, compared to HCs, patients with MDD showed higher nodal CC only in the right precuneus. These findings might imply that patients with BD could be associated with a more severely altered network. Our results are in line with some previous studies where MDD patients showed altered global network properties, while they presented almost the same nodal level network as that of HCs^23,33^. However, these studies only included patients with MDD. To verify our findings, further studies with both patient groups are necessary.

Previous studies have repeatedly shown an abnormality in the precuneus in both BD and MDD patients, which is in line with our results. BD patients showed a lack of precuneus activation during emotion related paradigms^41,42^. A resting-state fMRI study^43^ found increased neuronal synchronization in the right precuneus, left superior frontal gyrus, and right paracentral lobule, indicating that these areas might play an important role in the pathophysiology of BD. In terms of MDD, lower activity in the right precuneus was observed relative to the HCs^44,45^. Zhu et al.^46^ found decreased connectivity of the precuneus in patients with MDD compared to HCs. In addition, a recent fMRI study found that reduced strength of the right precuneus was associated with higher maladaptive rumination levels in patients with MDD^47^. Among the few studies directly comparing both BD and MDD patients, Liang et al.^48^ found that the regional homogeneity value of the precuneus was increased in BD and MDD patients. Another fMRI study^24^ found similar network changes between BD and MDD, but abnormal nodal efficiency in the right precuneus was specific to BD patients.

Moreover, evidence of abnormalities in other regions besides the precuneus has been accumulated in BD. For example, a diffusion-weighted MRI study^29^ revealed abnormalities of nodal network measures in the bilateral isthmus cingulate in BD. Another structural brain network study^49^ identified alterations in community structure containing the paracentral gyrus and posterior cingulate in BD. They also found that the left isthmus cingulate alteration was associated with the number of depressive episodes in patients with BD. With regard to the superior frontal region, cortical thinning or volume reduction in the left superior frontal cortex, a key emotional processing region, has been observed in BD patients^50,51^. A diffusion tensor imaging study reported reduced fractional anisotropy in the superior frontal white matter in BD patients^52^. Regarding the paracentral region, patients with BD showed increased gray matter volume in the bilateral paracentral lobule^53^. An fMRI study^54^ reported reduced nodal degree and increased participation coefficient in the paracentral lobule in BD. These studies support our findings that the regions could play a crucial role in comprehending the pathophysiology of BD patients. Furthermore, our findings might imply that the network of BD patients could be more functionally altered than that of patients with MDD.

Our findings showed the significant differences of nodal CCs in five regions among the three groups; right precuneus, left isthmus cingulate, left paracentral, right paracentral, and left superior frontal. For nodal CCs of the contralateral regions including left precuneus, right isthmus cingulate, and right superior frontal, they showed the similar trend with *p *values lower than 0.01. However, after applying Bonferroni corrections for multiple comparisons, they were not considered significant with an adjusted *p *value. Our findings are warranted to be further replicated from a larger number of patients considering various characteristics to verify if there are hemispheric differences in the changes of the nodal CCs.

Interestingly, our findings revealed intermediate network measures of patients with MDD, between those in patients with BD and those in HCs, although the two patient groups did not show significantly different network measures. Our BD patient population was composed of more BD Type II patients than BD Type I patients (25 patients with BD Type II and 10 patients with BD Type I). BD II is distinguished from BD I mainly by the absence of full-blown manic episodes, and a growing body of evidence suggests that there could be neurobiological differences between BD I and BD II patients^55^. Therefore, the higher proportion of patients with BD II in the present study might have affected our results that there were no significant differences in network measures between the two patient groups. Furthermore, the difference between BD and MDD patients might not be detected because the current study population did not include patients with psychotic symptoms. The comorbid psychotic features are usually related to low functioning and disability^56^ and worse cognitive performance in BD^57^. Further research needs to be conducted with a large sample size of both BD subtypes and various symptom dimensions, such as psychotic features.

Moreover, our significant correlation result was found only in BD patients where the nodal level high beta band CC of the left superior frontal region was positively correlated with state anxiety. One possible explanation for the result could be the more altered network in BD than MDD. In other words, although both patient groups did not show differences in phenotypic anxiety levels through self-reports, more altered network in the left superior frontal region in the patients with BD could have been more affected by anxiety. Superior frontal region has been regarded to be related to the attention network and DMN^58^. Attention network is thought to mediate goal directed top down processing which is associated with task control function. Brain regions belonging to attention network could play a role in emotional regulation and in the higher state of vigilance and awareness^58^. Considering the cognitive models of anxiety disorders, which emphasized the role of emotional hyperactivity^59^, a failure of emotional regulation is thought to be an essential cause of anxiety symptoms in mood disorder^60^. In addition, increased connectivity in the DMN is associated with ruminative thoughts and excessive worries during self-referential processing^61^. Thus, the link between nodal CC in the DMN-related region and state anxiety suggests more negative internalized attention in patients with BD^62^. Furthermore, the high beta band frequency domain has been shown to be related to anxiety^63,64^. The altered attention network and DMN activity in the beta frequency band was repeatedly observed in patients with BD^34–36^. Taken together, the disrupted beta band network in superior frontal region in BD patients might contribute to affective functioning underlying clinical symptoms such as anxiety.

This study has a few limitations. First, some of the patients were taking medications at the time of testing. However, less than 30% of the patients were taking medications in each group (9 patients with BD and 11 patients with MDD). Further studies controlling for the medication effects would be needed. Second, the current study enrolled patients with depression but without psychotic symptoms. Thus, our findings may not be generalized to the entire population of individuals with BD and MDD. Third, we did not use individual head models for EEG source imaging. Source analysis of scalp-derived EEG might be inherently limited because of the precision of spatial localization. Despite these limitations, this study was the first attempt to investigate and compare source-level cortical functional networks in patients with BD and MDD during resting-state EEG. Our results suggest that both patients have similar network changes at the global level, but they have different network changes at the nodal level. Also, the higher nodal CCs in the high beta band might indicate the regions became more connected with their neighbors in accordance with the severity of depressive and anxious states. We also found a significant correlation between cortical network state and anxiety-related psychological measure in BD patients. Our source-level cortical network indices might contribute to the understanding of the neuropathological mechanisms in these two disorders. Further studies with larger sample sizes are necessary to replicate and extend the generalizability of our findings.

## Materials and methods

### Participants

Participants were recruited from the Department of Psychiatry at Soonchunhyang University Cheonan Hospital in Korea. The patients included 35 patients with BD (14 men and 21 women; mean age: 32.57 ± 11.61 years; range: 19–56 years) and 39 patients with MDD (20 men and 19 women; mean age: 31.54 ± 12.32 years; range: 19–59 years). Patients with BD and MDD were diagnosed according to the Structured Clinical Interview for Diagnostic and Statistical Manual of Mental Disorders, 4th edition (DSM-IV) Axis I Psychiatric Disorders by a board-certified psychiatrist^65^. In addition, the board-certified psychiatrist confirmed that the patients with BD had one or more hypomanic/manic episodes in their lifetime mood disorders. Patients with any history of neurological or other severe medical diseases were excluded in the initial screening interviews. None of the patients had mental retardation, alcohol abuse, were undergoing electroconvulsive therapy, or had any head injuries. Among the 35 patients with BD, 10 patients were diagnosed with BD Type I and 25 patients with BD Type II. Nine of the 35 patients with BD were taking mood-stabilizing agents (lithium and valproate) with or without atypical antipsychotics (quetiapine, aripiprazole, and olanzapine). Eleven of the 39 patients with MDD were taking medications, such as selective serotonin reuptake inhibitors (fluoxetine and escitalopram), serotonin, norepinephrine reuptake inhibitors (duloxetine), or others (mirtazapine). Forty-two HCs (23 men and 19 women; mean age: 29.43 ± 5.95 years; range: 21–41 years) were recruited through posters in the hospital and advertisements in local newspapers. An initial screening interview was conducted by a board-certified psychiatrist to exclude any subjects with identifiable psychiatric disorders, neurological disorders, or histories of head injuries. All participants were right-handed. This study was approved by the Institutional Review Board and Ethics Committee of Soonchunhyang University Cheonan Hospital, and all experimental protocols were approved by the committee (2018-10-032-006). The study was performed in accordance with approved guidelines. Informed consent was acquired from all study participants.

### Psychological measures

The State-Trait Anxiety Inventory (STAI)^66,67^ and Beck Depression Inventory (BDI)^68^ were evaluated for anxiety and depression. The STAI is a self-rating scale for state and trait anxiety^67^. It consists of a state anxiety inventory and trait anxiety inventory; each inventory has 20 items^66^. The BDI is a self-rating scale composed of 21 items to measure the severity of depression symptoms^68^.

### Recording and preprocessing of electroencephalography (EEG)

Resting-state EEG data were recorded in a sound-attenuated room, while the participants opened their eyes for five minutes. EEG was recorded with a NeuroScan SynAmps2 amplifier (Compumedics USA, Charlotte, NC, USA) based on an extended 10–20 placement scheme via 62 Ag–AgCl electrodes mounted on a Quik-Cap. The reference electrode was Cz and the ground electrode was located on the forehead. Horizontal electrooculogram (EOG) electrodes were placed at the outer canthus of each eye while vertical EOG electrodes were located above and below the left eye. The impedance was kept below 5 kΩ. EEG signals were bandpass filtered from 0.1 to 100 Hz with a 1000-Hz sampling rate. The procedure for the EEG recording followed our previous study^20^.

All preprocessing procedures were performed using CURRY 8 (Compumedics USA, Charlotte, NC, USA) and MATLAB R2018b (MathWorks, Natick, MA, USA). The EEG data were re-referenced to an average reference. In order to remove DC components, a high pass filter with 1 Hz was applied to the EEG data. Visual inspection for movement artifacts was carried out by a skilled researcher without prior information regarding the data origin. Eye-movement related artifacts were corrected via a covariance- and regression-based mathematical procedure in CURRY 8^69^. Then, the preprocessed EEG data were divided into 2.048 s (2048 points) epochs, and all the epochs including significant physiological artifacts (amplitude exceeding ± 100 μV) at any of the 62 electrodes were rejected. After all the preprocessing procedures, the three groups did not show a significant difference regarding the number of artifact-free epochs (patients with BD: 125.00 ± 31.99; patients with MDD: 119.56 ± 37.71; HCs: 108.62 ± 29.93; *p* = 0.091). Among the remaining artifact-free epochs, 30 epochs were randomly extracted for each participant considering the bias over time. Finally, each participant had 30 artifact-free epochs. It was determined by the different number of remaining epochs from each participant after rejecting artifacts. In addition, a previous study demonstrated acceptable reliability of resting-state EEG data longer than 40 s^70^.

### Source localization

The depth-weighted minimum L2 norm estimator from the Brainstorm toolbox (http://neuroimage.usc.edu/brainstorm) was used to approximate the time series of source activities^71^. A three-layer boundary element model from the MNI/Colin 27 anatomy template was used to compute the lead-field matrix. Cortical current density of 15,002 cortical vertices was calculated at every time point of each epoch. Noise covariance was measured by the whole 30 epochs of each participant. The weight of each individual sensor was computed by the diagonal components of the noise covariance for the source reconstruction. After approximating the cortical current density at every time point, 68 nodes were extracted based on the Desikan–Killiany atlas containing 34 cortical regions in each hemisphere^72^. The Brainstorm toolbox provided the information about which cortical vertex belonged to which region for 15,002 cortical vertices based on the atlas. The representative value in each region was assessed by the cortical source of the seed point in each region based on the Desikan–Killiany atlas, which information was provided in the Brainstorm toolbox. Then, the time series of the cortical sources at each of the 68 seed points were bandpass filtered and divided into six frequency bands: delta (1–4 Hz), theta (4–8 Hz), alpha (8–12 Hz), low beta (12–18 Hz), high beta (18–30 Hz), and gamma (30–55 Hz). The procedure for the source localization followed that of our previous study^20^.

### Connectivity and network analysis

Functional connectivity between each pair of nodes was assessed via PLVs^73^. PLVs provide normalized synchronization values ranging from 0 to 1. Thus, no further modification was needed to apply them to the weighted network analysis. A higher PLV denotes stronger connection between two nodes than that between the other pairs. PLV showed fine performance with weighted minimum norm estimation^74^ and has been widely employed in network analysis^75,76^.

We performed a graph theory-based weighted network analysis^17,18^. The weighted network preserves the unique traits of the original network without distortion. A network is comprised of several nodes connecting to each other using edges. Widely used three representative global level weighted network measures were evaluated in this study. First, “strength” denotes the degree of connection strength in the network. A higher strength value indicates the strong connection in the whole brain. Second, “CC” refers to the degree to which a node clusters with its neighboring nodes. An increased CC denotes a well-segregated network between the relevant brain regions. Third, “PL” denotes the sum of lengths between two nodes within the network. It is associated with the information processing speed. The shortened PL refers to a well-integrated network. Furthermore, weighted nodal CC was assessed at each node. Network analyses were carried out via MATLAB R2018b.

### Statistical analysis

Chi-squared tests and one-way analysis of variance (ANOVA) were used to explore differences in demographic characteristics and psychological measures among the three groups. A multivariate ANOVA was conducted to compare the cortical network characteristics at the global level of each frequency band among the three groups, with education as a covariate. *P *values were adjusted by Bonferroni corrections (an adjusted *p *value of 0.05/18 = 0.002778; three global network measures with six frequency bands) to control multiple comparisons. The same analysis was conducted at the nodal level, followed by Bonferroni corrections with an adjusted *p *value of 0.05/68 = 0.000735 (nodal CCs at 68 nodes). When the variables presented significant differences among the three groups, the post-hoc pair-wise comparisons with Bonferroni corrections were performed. Effect sizes were computed based on the partial eta squared (η^2^). The procedure for the statistical analysis followed our previous study^20^.

The relationship between network indices and psychological measures was analyzed using hierarchical regression analyses with a 5000 bootstrap resampling technique to correct for multiple comparisons. For the patient groups, the duration of illness was considered as a covariate. Although the bootstrap test is weaker than the Bonferroni test to resolve the multiple-comparison issue, the robustness and stability of the bootstrap test have been verified^77,78^. Furthermore, the bootstrap test has been widely used in EEG analysis^79,80^. Statistical analyses were performed using SPSS 21 with the significant level at *p* < 0.05 (two-tailed) (SPSS, Inc., Chicago, IL, USA).

## Supplementary Information

Supplementary Information.
