# Supplementary material for: Alteration of cortical functional networks in mood disorders with resting-state electroencephalography
Source: Sci Rep. 2022 Apr 8;12:5920. doi: 10.1038/s41598-022-10038-w (PMC8993886; doi:10.1038/s41598-022-10038-w)
Supplement: Supplementary file 1 — Supplementary Information. [file 41598_2022_10038_MOESM1_ESM.docx]

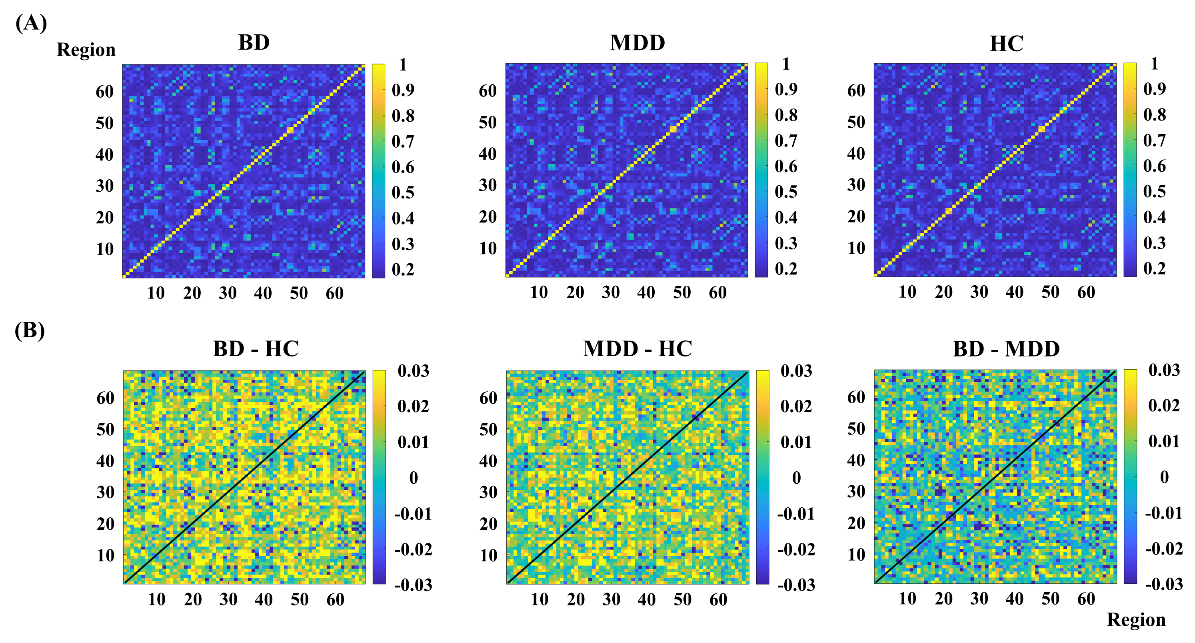


Supplementary Figure. (A) mean weighted matrices calculated from phase-locking values for the high beta band in each group. (B) differences of mean weighted matrices between each pair of two groups for the high beta band.
